# Ancient lineages of arbuscular mycorrhizal fungi provide little plant benefit

**DOI:** 10.1007/s00572-021-01042-5

**Published:** 2021-07-30

**Authors:** Verena Säle, Javier Palenzuela, Concepción Azcón-Aguilar, Iván Sánchez-Castro, Gladstone Alves da Silva, Benjamin Seitz, Ewald Sieverding, Marcel G. A. van der Heijden, Fritz Oehl

**Affiliations:** 1 Plant-Soil-Interactions, Agroscope, Reckenholzstrasse 191, CH-8046 Zürich, Switzerland; 2 Department of Evolutionary Biology and Environmental Studies, University of Zürich, Winterthurerstrasse 190, CH-8057 Zürich, Switzerland; 3 Vegetable-Production Extension, Agroscope, Müller-Thurgau-Strasse 29, CH-8820 Wädenswil, Switzerland; 4 Departamento de Microbiología del Suelo Y Sistemas Simbióticos, Estación Experimental del Zaidín, CSIC, Profesor Albareda 1, 18008 Granada, Spain; 5 Departamento de Microbiología, Universidad de Granada, Campus Universitario de Fuentenueva, 18071 Granada, Spain; 6 Departamento de Micologia, CCB, Universidade Federal de Pernambuco, Av. da Engenharia s/n, Cidade Universitária, Recife, PE 50740-600 Brazil; 7 Institute of Agricultural Sciences in the Tropics (Hans-Ruthenberg Institute), University of Hohenheim, Garbenstr. 13, 70599 Stuttgart-Hohenheim, Germany; 8 Department of Plant and Microbial Biology, University of Zürich, 8057 Zürich, Switzerland; 9 Ecotoxicology, Agroscope, Müller-Thurgau-Strasse 29, CH-8820 Wädenswil, Switzerland

**Keywords:** Arbuscular mycorrhizal fungi (AMF), Evolution, Functional diversity, Phylogeny, Plant benefit

## Abstract

Almost all land plants form symbiotic associations with arbuscular mycorrhizal fungi (AMF). Individual plants usually are colonized by a wide range of phylogenetically diverse AMF species. The impact that different AMF taxa have on plant growth is only partly understood. We screened 44 AMF isolates for their effect on growth promotion and nutrient uptake of leek plants (*Allium porrum*), including isolates that have not been tested previously. In particular, we aimed to test weather AMF lineages with an ancient evolutionary age differ from relatively recent lineages in their effects on leek plants. The AMF isolates that were tested covered 18 species from all five AMF orders, eight families, and 13 genera. The experiment was conducted in a greenhouse. A soil–sand mixture was used as substrate for the leek plants. Plant growth response to inoculation with AMF varied from − 19 to 232% and depended on isolate, species, and family identity. Species from the ancient families Archaeosporaceae and Paraglomeraceae tended to be less beneficial, in terms of stimulation plant growth and nutrient uptake, than species of Glomeraceae, Entrophosporaceae, and Diversisporaceae, which are considered phylogenetically more recent than those ancient families. Root colonization levels also depended on AMF family. This study indicates that plant benefit in the symbiosis between plants and AMF is linked to fungal identity and phylogeny and it shows that there are large differences in effectiveness of different AMF.

**Supplementary Information:**

The online version contains supplementary material available at 10.1007/s00572-021-01042-5.

## Introduction

Arbuscular mycorrhizal fungi (AMF) play a key role in ecosystems and promote plant growth and nutrition (Smith and Read 2008; van der Heijden et al. 2015). AMF are abundant in almost all natural soils and have been considered as keystone taxa in soil microbial communities (Banerjee et al. 2019). AMF are able to establish symbioses with the majority of terrestrial plants. In return for photosynthates, the fungi provide several benefits to their host plants. These include increased phosphorus and nitrogen uptake and enhanced resistance to drought and root pathogens (Jia et al. 2020; Smith and Read 2008; van der Heijden et al. 2015) as well as mitigation of salinity stress (e.g., Evelin et al. 2019). Also, improved soil quality by aggregating soil particles repeatedly has been reported (e.g., Rillig and Mummey 2006). In addition, diverse AMF communities can lead to elevated diversity and productivity of plant communities (Jansa et al. 2008; van der Heijden et al. 1998a). Hence, crop production can profit from these positive effects which might be expressed in higher yield or reduced use of phosphatic fertilizer. The latter is advantageous in view of the growing demand for sustainable strategies in agricultural systems (Bender et al. 2016; Sorensen et al. 2008).

The effectiveness of the symbiosis depends, among other factors, on the combination of plant and AM fungus, soil type, soil fertility, and the origin of the AMF isolates (de Novais et al. 2014; Feddermann et al. 2010; Pringle and Bever 2008). It has been assumed for instance that AMF isolated from polluted sites are advantageous for the bioremediation of such sites (e.g., Cabello 1999; Hildebrandt et al. 2007; Takács 2012), or indigenous AMF isolates are superior to exotic isolates for plant growth promotion (e.g., Estrada et al. 2013; Klironomos 2003; Oliveira et al. 2005; Tchabi et al. 2010). Furthermore, high functional diversity among different AMF species and even among isolates belonging to the same species (Feddermann et al. 2010; Munkvold et al. 2004) has been observed. Because of this high functional diversity, it is important to investigate a wide range of different plant–AMF combinations (Feddermann et al. 2010). For example, some AMF taxa are most efficient to supply nutrients to their host plants, while others provide notable resistance against biotic or abiotic stresses (Fester and Sawers 2011; Verbruggen et al. 2010). Functional traits may be phylogenetically conserved, i.e., symbiotic functions such as plant growth promotion depend on AMF lineages (Powell et al. 2009). A meta-analysis from Hoeksema et al. (2018), however, suggested that AMF phylogeny is not related to plant response; instead, recent diversification among plants explains variation in the arbuscular mycorrhizal symbiosis.

Previous studies have shown that different AMF families and clades significantly affect plant biomass, with some clades having superior effects on host plants, while others have no or weak effects (e.g., Hart and Reader 2002a; Sieverding et al. 2014). For example, van der Heijden et al. (1998a) and Powell et al. (2009) observed that the ancient AMF (e.g., *Paraglomus* (isolate BEG 21 in van der Heijden et al. 1998a) and *Archaeospora* (in Powell et al. 2009) are less beneficial in promoting plant growth than more recently evolved AMF including *Rhizoglomus* and *Funneliformis*. Nevertheless, so far, it is unclear whether symbiotic benefit is linked to the phylogenetic distances among AMF species and whether those distances can be used to make predictions regarding the growth-promoting properties of AMF. This issue deserves increased attention, especially because there are only a few studies of members of the Paraglomerales and Archaeosporales, despite their widespread occurrence in many ecosystems (Davison et al. 2015). There is one study from Koch et al. (2017) that tested three host plants and 56 isolates, including several members of ancient AMF families (e.g., *Archaeospora trappei* and *Paraglomus occultum*). Koch et al. (2017) conclude that host performance cannot be predicted from AM fungus morphology and growth traits. Rather divergent effects on plant growth among isolates within an AM fungus species may be caused by coevolution between co-occurring fungus and plant populations.

The major objective of the present study was to investigate how AMF taxonomic levels and phylogeny affect plant growth and nutrient uptake. In order to test this, we compared 44 AMF isolates, comprising all five glomeromycotan orders, eight of 16 AMF families, and 13 of 48 AMF genera, including several taxa not tested previously in systematic investigations (e.g., *Rhizoglomus invermaium*, *Dominikia compressa*, and *Cetraspora helvetica*). The 44 isolates all had been prepared under similar conditions and were propagated on *Hieracium pilosella* L. on the same soil–sand substrate for 1 year before screening them on leek. These isolates were characterized morphologically and molecularly using the partial large subunit (LSU) of the ribosomal gene after DNA extraction from spores. We assessed the impact of those different isolates on growth and plant nutrient concentrations of leek (*Allium porrum* L.) for different levels of taxa, i.e., on the levels of isolates, species, genera, and families. Specifically, we tested whether (i) plant growth and root colonization differs depending on AMF taxa and AMF families; (ii) members of ancient AMF families differed in their effects on plant growth compared to more recently evolved AMF families; and (iii) the phylogenetic placement of AMF isolates could explain their impacts on plant growth. We hypothesized that leek growth as well as nutrient assimilation and root colonizing strategies differ among AMF isolates and species, and even among higher taxa and major phylogenetic clades. In addition, we hypothesize that ancient AMF are less mutualistic in terms of plant growth stimulation than relatively recently evolved AMF families.

## Materials and methods

### AMF isolates for screening effects on leek growth

Between July 2011 and June 2012, 150 monosporal and 90 multisporal cultures were grown on *H. pilosella* in the Swiss collection for arbuscular mycorrhizal fungi (SAF) (https://www.agroscope.ch/saf), as described in Tchabi et al. (2010). The substrate for AMF propagation was a mixture of three parts Terragreen (American aluminum oxide, Oil Dry US special, type III R) and one part Loess with the following parameters: pH-H_2_O 6.8; organic carbon 3.0 g kg^−1^; available P (P-double lactate) 15.6 mg kg^−1^; available K (Na-acetate) 350 mg kg^−1^.

Besides being of monosporal or multisporal origin, all isolates were propagated identically in the glasshouse. After initial propagation, the inocula were air-dried and stored for 12 months before inoculation to leek (*Allium porrum* L.). Forty-four AMF isolates were selected from the SAF for the screening experiment on leek, comprising 18 AMF species, 13 genera, eight families, five orders, and three AMF classes. Per AMF species, one to four isolates were selected, whenever possible deriving from different isolation sites (Table 1). Except the two *Scutellospora calospora* isolates, which were derived from soils in southwestern Germany, all isolates originated from Swiss soils. Thirty-five isolates (“11-FO…” isolates in Table 1) were from monosporal AMF cultures, established as described in Tchabi et al. (2010). Two other isolates (of *Sc. calospora*) were derived from one monosporal culture established in 2001 (“01-FO…” isolates) and were propagated in two separate pots between July 2011 and June 2012 especially for the leek screening experiment. Seven additional isolates (one of *Diversispora celata*, and two each of *Gigaspora margarita*, *Cetraspora helvetica*, and *Paraglomus laccatum*) were derived from multiple spore cultures originally established in 1994–2002 (see Oehl et al. 2010, 2014a, b; van der Heijden et al. 1998b) and were propagated for our purposes as mentioned above for *Sc. calospora*. The inocula were checked for their spore densities. To create equal preconditions for the non-mycorrhizal treatment, the inoculum for the control derived from an unsuccessful monosporal AMF culture on *H. pilosella*, in which no AMF symbiosis could be established and no AMF spores had been formed.

**Table 1:** List of AMF isolates used in this study together with reference collection numbers (SAF = Swiss collection of arbuscular mycorrhizal fungi; original accession number) and information on the original isolation sites of the AMF isolates. Except two isolates from Germany, all other isolates originated from soils in Switzerland

Order	Isolate	SAF accession	Original accession	NCBI GenBank accession	Village and canton of origin in Switzerland	Land use at origin site	Soil pH	In pure culture since	Soil type^1^ at origin
Family									
Species									
Glomerales									
Glomeraceae									
*Oehlia diaphana*	O.dia1	SAF106	11-FO106	MN996942	Uettlingen BE	Arable Field (Winter Wheat)	5.3	2011	Eutric Cambisol
*Oehlia diaphana*	O.dia2	SAF107	11-FO290	MN996943	Graswil BE	Arable field (winter barley)	5.6	2011	Eutric Cambisol
*Oehlia diaphana*	O.dia3	SAF108	11-FO292	MN996944	Graswil BE	Arable field (winter barley)	5.6	2011	Eutric Cambisol
*Rhizoglomus irregulare*	R.irr1	SAF130	11-FO113	MN996945	Uettlingen BE	Arable field (winter wheat)	5.3	2011	Haplic Luvisol
*Rhizoglomus irregulare*	R.irr2	SAF131	11-FO190	MN996946	Frick AG	Arable field (winter wheat)	7.6	2011	Vertic Cambisol
*Rhizoglomus irregulare*	R.irr3	SAF170	11-FO420	MN996947	Langnau BE	Permanent grassland	5.5	2011	Eutric Cambisol
*Rhizoglomus irregulare*	R.irr4	SAF96	11-FO181	MN996948	Frick AG	Arable field (winter wheat)	7.6	2011	Vertic Cambisol
*Rhizoglomus invermaium*	R.inv1	SAF205	11-FO84	LN624111-12	Hindelbank BE	Arable field (grass–clover)	7.1	2011	Eutric Cambisol
*Rhizoglomus invermaium*	R.inv2	SAF206	11-FO424	MN996965	Langnau BE	Permanent grassland	5.5	2011	Eutric Cambisol
*Rhizoglomus invermaium*	R.inv3	SAF207	11-FO432		Rubigen BE	Permanent grassland	5.8	2011	Eutric Cambisol
*Rhizoglomus invermaium*	R.inv4	SAF147	11-FO336	MN996959	Rubigen BE	Permanent grassland	5.8	2011	Eutric Cambisol
*Funneliformis mosseae*	F.mos1	SAF87	11-FO85	MN996949	Hindelbank BE	Arable field (grass–clover)	7.1	2011	Haplic Luvisol
*Funneliformis mosseae*	F.mos2	SAF139	11-FO239	MN996950	Graswil BE	Arable field (winter barley)	5.6	2011	Haplic Luvisol
*Funneliformis mosseae*	F.mos3	SAF160	11-FO418	MN996951	Langnau BE	Permanent grassland	5.5	2011	Eutric Cambisol
*Funneliformis caledonius*	F.cal	SAF111	11-FO269	MN996952	Graswil BE	Arable field (winter barley)	5.6	2011	Haplic Luvisol
*Funneliformis fragilistratus*	F.fra1	SAF109	11-FO185		Frick AG	Arable field (winter wheat)	7.6	2011	Vertic Cambisol
*Funneliformis fragilistratus*	F.fra2	SAF110	11-FO193	MN996960	Frick AG	Arable field (winter wheat)	7.6	2011	Vertic Cambisol
*Septoglomus nigrum*	Se.nig1	SAF86	11-FO61	MK234700	Niederösch BE	Permanent grassland	5.7	2011	Haplic Luvisol
*Septoglomus nigrum*	Se.nig2	SAF175	11-FO471	MK234701	Rubigen BE	Arable field (winter barley)	7.1	2011	Eutric Cambisol
*Dominikia compressa*	Do.com1	SAF145	11-FO332		Rubigen BE	Permanent grassland	5.8	2011	Eutric Cambisol
*Dominikia compressa*	Do.com2	SAF203	11-FO352	HG798895-99	Rubigen BE	Permanent grassland	5.8	2011	Eutric Cambisol
Entrophosporaceae (late synonym Claroideoglomeraceae^2^)									
*Claroideoglomus candidum*	Cl.can	SAF112	11-FO411	MN996953	Langnau BE	Permanent grassland	5.5	2011	Eutric Cambisol
*Claroideoglomus claroideum*	Cl.cla1	SAF92	11-FO55	MN996961	Niederösch BE	Permanent grassland	5.7	2011	Haplic Luvisol
*Claroideoglomus claroideum*	Cl.cla2	SAF181	11-FO94		Hindelbank BE	Permanent grassland	7.1	2011	Haplic Luvisol
*Claroideoglomus claroideum*	Cl.cla3	SAF166	11-FO370		Bantigen BE	Arable field (grass–clover)	6.2	2011	Haplic Luvisol
*Entrophospora infrequens*	E.inf1	SAF209	11-FO321		Bantigen BE	Arable field (grass–clover)	6.2	2011	Eutric Cambisol
*Entrophospora infrequens*	E.inf2	SAF210	11-FO313		Bantigen BE	Arable field (grass–clover)	6.2	2011	Eutric Cambisol
Diversisporales									
Diversisporaceae									
*Diversispora celata*	Di.cel1	SAF5	HG-234	MN996954	Eschikon ZH	Permanent grassland	7.0	2002^3^	Haplic Luvisol
*Diversispora celata*	Di.cel2	SAF151	11-FO387	MN996955	Bantigen BE	Permanent grassland	5.3	2011	Haplic Luvisol
*Diversispora celata*	Di.cel3	SAF152	11-FO403	MN996956	Langnau BE	Permanent grassland	5.5	2011	Haplic Luvisol
*Diversispora epigaea*	Di.epi1	SAF118	11-FO459	MN996962	Rubigen BE	Arable field (winter barley)	7.1	2011	Eutric Cambisol
*Diversispora epigaea*	Di.epi2	SAF128	11-FO338	MN996963	Rubigen BE	Permanent grassland	5.8	2011	Eutric Cambisol
*Diversispora epigaea*	Di.epi3	SAF129	11-FO460	MN996964	Rubigen BE	Arable field (winter barley)	7.1	2011	Eutric Cambisol
Gigasporales									
Gigasporaceae									
*Gigaspora margarita*	G.mar1	SAF14-1	JJ-4		Tänikon TG	Arable field	6.2	2000^3^	Haplic Luvisol
*Gigaspora margarita*	G.mar2	SAF14-2	JJ-4		Tänikon TG	Arable field	6.2	2000^3^	Haplic Luvisol
Racocetraceae									
*Cetraspora helvetica*	Ce.hel1	SAF15-1	JJ17/19	HM565946	Tänikon TG	Arable field	6.2	2000^3^	Haplic Luvisol
*Cetraspora helvetica*	Ce.hel2	SAF15-2	JJ17/19	HM565945	Tänikon TG	Arable field	6.2	2000^3^	Haplic Luvisol
Scutellosporaceae									
*Scutellospora calospora*	Sc.cal1	SAF202-1	01-FO30	MN996957	Vogtsburg, Germany	Vineyard	7.7	2001^4^	Eutric Cambisol
*Scutellospora calospora*	Sc.cal2	SAF202-2	01-FO30		Vogtsburg, Germany	Vineyard	7.7	2001^4^	Eutric Cambisol
Archaeopsporales									
Archaeosporaceae									
*Archaeospora europaea*	A.eur1	SAF113	11-FO107		Uettlingen BE	Arable field (winter wheat)	5.3	2011	Eutric Cambisol
*Archaeospora europaea*	A.eur2	SAF114	11-FO126		Frick AG	Arable field (winter wheat)	7.6	2011	Vertic Cambisol
*Archaeospora europaea*	A.eur3	SAF115	11-FO345		Rubigen BE	Permanent grassland	5.8	2011	Eutric Cambisol
Paraglomerales									
Paraglomeraceae									
*Paraglomus laccatum*	P.lac1	SAF56-1	BEG21	MN996958	Nenzlingen BL	Permanent grassland	7.7	1994^5^	Calcaric Leptosol
*Paraglomus laccatum*	P.lac2	SAF56-2	BEG21		Nenzlingen BL	Permanent grassland	7.7	1994^5^	Calcaric Leptosol

^1^According to IUSS Working Group WRB (2015)

^2^Taxa names are given based on nomenclatural rules. Here, we follow nomenclature after Oehl et al. (2011a, b), updated in Baltruschat et al. (2019) and Wijayawardene et al. (2020). The nomenclature of AMF is still partly under debate and some AMF are named differently by different authors (e.g., Krüger et al. 2012; Wijayawardene et al. 2020)

^3^Deposited at SAF 2008

^4^Propagated 2011/2012 for this study

^5^Pure culture in 1994; re-established in 2008

### Morphological characterization of AMF isolates

Spores of the 44 AMF isolates were extracted from the inocula substrates by wet sieving and decanting followed by sucrose gradient centrifugation and counted in Petri dishes under a dissecting microscope as described in Sieverding (1991). The spores were morphologically identified from specimens mounted in polyvinyl-lacto-glycerol (PVLG; Koske and Tessier 1983) and a mixture of PVLG and Melzer’s reagent (Brundrett et al. 1994). Reference slides of the isolates were deposited at the mycological herbarium of ETH Zurich (Z + ZT). For morphological spore identification, the identification manual of Błaszkowski (2012) for Glomeromycota was used and was based on the classification system of Oehl et al. (2011b), considering the most recent updates (e.g., Baltruschat et al. 2019; Błaszkowski et al. 2015, 2018a, b; Błaszkowski et al. 2019; Wijayawardene et al. 2020).

The identifications of the 44 AMF isolates resulted in 18 species from eight families. The isolates from the order Glomerales were most numerous and belonged to the families Glomeraceae and Entrophosporaceae (Table 1).

### Molecular characterization of the AMF isolates

A subset of the AMF spores extracted was used for molecular analyses as described in Palenzuela et al. (2015). Five spores were isolated from each AMF inoculum originating from propagations on *H. pilosella* in the glasshouses of Agroscope in Zurich-Reckenholz. Spores were surface-sterilized with chloramine T (2%) and streptomycin (0.02%; Mosse 1962), and all five together were crushed with a sterile disposable micropestle in 23 µl milli-Q water as described in Palenzuela et al. (2013). Deoxyribonucleic acid (DNA) amplification of the crude extracts was performed in an automated thermal cycler (Gene Amp PCR System 2400, Perkin-Elmer, Foster City, California) using pureTaq Ready-To-Go PCR Beads (Amersham Biosciences Europe GmbH, Germany) by following the manufacturer’s instructions. A two-step polymerase chain reaction (PCR) was conducted to increase the specificity of amplification. A ∼ 1500-bp fragment was amplified comprising the SSU end, ITS1, 5.8S, ITS2, and partial LSU rDNA using the SSUmAf/LSUmAr and SSUmCf/LSUmBr primers consecutively (Krüger et al. 2009; Oehl et al. 2019a, b). PCR products were analyzed by electrophoresis in 1.2% agarose gels stained with Gel Red™ (Biotium Inc., Hayward, CA, USA) and viewed by UV illumination. Amplicons of the expected size were purified using the Illustra GFX PCR DNA and Gel Band Purification kit and were directly sequenced. For those for which not fair sequences were obtained, a portion of the purified PCR product was cloned into the PCR 2.1 vector (Invitrogen, Carlsbad, CA, USA), and transformed into One shot© TOP10 chemically competent *Escherichia coli* cells. After plasmid isolation from transformed cells, the cloned DNA fragments were sequenced with vector primers (White et al. 1990) in both directions by Taq polymerase cycle sequencing on an automated DNA sequencer (Perkin-Elmer ABI Prism 373).

DNA sequences are deposited in the NCBI GenBank (www.ncbi.nlm.nih.gov/genbank/); accession numbers are given in Table 1.

### Phylogenetic analyses

The phylogeny was reconstructed by analyses of the partial LSU rDNA. The AM fungal sequences obtained were aligned with other Glomeromycota sequences from GenBank in ClustalX2 (Larkin et al. 2007). *Boletus edulis* Bull. and *Mortierella ambigua* B. S. Mehrotra were included as an outgroup. Prior to phylogenetic analysis, the model of nucleotide substitution was estimated using Topali 2.5 (Milne et al. 2004). Bayesian analysis (two runs over 3 × 10^6^ generations with a sample frequency of 300 and a burn-in value of 25%) was performed in MrBayes 3.1.2 (Ronquist and Huelsenbeck 2003), launched from Topali 2.5, using the GTR + G model. Our annotations follow the most recent systematics of the Glomeromycota (e.g., Baltruschat et al. 2019; Błaszkowski et al. 2018a, b; Corazon-Guivin et al. 2019; Oehl et al. 2019a; Silva et al. 2012; Tedersoo et al. 2018). Table 1 gives detailed information about sequence origin.

Isolates A.eur1, A.eur2, A.eur3, R.inv3, F.fra1, Do.com1, Cl.cla2, Cl.cla3, E.inf1, E.inf2, G.mar1, and G.mar2 were not included in the phylogenetic analyses because no DNA sequences could be obtained.

### Experiment setup

To test the effect of the different AMF isolates on leek growth promotion and leek macronutrient uptake, a pot experiment was established in a glasshouse. In addition to the 44 AMF isolates (see Table 1), a non-mycorrhizal control was included. Each treatment was replicated six times, resulting in a total of 270 pots. As substrate, a mixture (1:1) of Loess subsoil which had been passed through a 5 mm sieve before autoclaving and mixing, and quartz sand was used. Soil and sand were separately sterilized by autoclaving (121 °C, 90 min) and afterwards mixed in equal proportion by weight. The chemical parameters of the substrate were as follows: pH (H_2_O) = 6.0, C_org_ = 1.4 g kg^−1^, P = 8.3 mg kg^−1^, potassium (K) = 31.5 mg kg^−1^, Ca = 910 mg kg^−1^, Mg = 149 mg kg^−1^. Parameters were measured according to standard methods in the laboratory of F.M. Balzer, Wetter-Amönau, Germany. Phosphorus (P), potassium (K), and magnesium (Mg) were extracted with double lactate according to the method of Hoffmann (1991). In that method, plant available nutrients are extracted from soil with 0.02 M Ca-lactate and 0.02 M hydrochloric acid. Calcium (Ca) was extracted with HCl and H_2_SO_4_. Each pot was filled with 500 g of the substrate and watered to 100% water capacity. For the AMF treatments, 5 ml inoculum was placed in a small, shallow hole in the center of each pot. The inoculum contained spores, root fragments, and substrate from the pot cultures of the AMF isolates. The control treatment received 5 ml of the same substrate, in which no AMF symbiosis was established during AMF inoculum propagation. The inocula were covered with a thin layer of substrate and ten seeds of leek (*Allium porrum* L., variety ‘Belton’, F1 hybrid) were sown exactly above the inocula. Seedlings were thinned after emergence to four plants per pot. The plants were maintained in the glasshouse under natural light and average temperatures of 25 °C by day and 18 °C at night. To ensure equal growth conditions, pots were completely randomized every 3 to 4 days. Plants were fertilized 3 weeks after emergence with 25 mg N, 10 mg P, and 25 mg K per pot. Aboveground biomass of leek plants was harvested 8 weeks after emergence to avoid plants becoming pot-bound.

### Leek biomass and nutrient analyses

Aboveground biomass was oven dried at 60 °C for 48 h and afterwards weighed. Before nutrient analyses, the dry matter was ground with a ball mill. Carbon and nitrogen concentrations of the shoots were determined with a CHNS-O Elemental Analyzer (Euro EA 3000, EuroVector SpA, Milan, Italy). For phosphorus, potassium, calcium, and magnesium, leek samples were dry ashed, solubilized in hydrochloric acid and subsequently measured with inductively coupled plasma optical emission spectrometry (Arcos FHS 16, Spectro Analytical Instruments GmbH, Kleve, Germany).

### AMF root colonization

To measure AMF root colonization, all leek roots were washed, stained with a 5% ink vinegar solution and mounted on microscope slides (Vierheilig et al. 1998). Hyphal, vesicular, and arbuscular root colonization were estimated according to the intersection method of McGonigle et al. (1990) using 100 intersections per sample.

### Statistical analyses

The total root length colonized and the formation of vesicles and arbuscules were analyzed by using a beta regression model suitable for rates with the link function probit. For the effects of AMF isolates, species and genera on leek biomass one-way analyses of variance (ANOVA) were performed, each followed by Dunnett’s test. To estimate the variance among species and of isolates within species, we used the restricted maximum likelihood (REML) method. When AMF family and order were used as explanatory variables, assumptions for an ANOVA—i.e., normal distribution of residuals and homogeneity of variances—could not be met; therefore, Kruskal–Wallis tests followed by Conover’s many-to-one test were applied. For the response variables P, N, K, Ca, and Mg concentrations in plant tissues, MANOVA was not possible; therefore, again Kruskal–Wallis and Conover’s many-to-one tests were run to compare AMF isolates. All significance levels were set at *p* < 0.05. In every post hoc test (Dunnett’s tests and Conover’s many-to-one tests), *p* values were adjusted according to the Benjamini–Hochberg procedure to correct for multiple testing (Benjamini and Hochberg 1995). The statistical analyses and graphing were carried out with the software R 4.0.5 (R Core Team 2021) using the packages stats, graphics, ggplot2 (Wickham 2016), betareg (Zeileis et al. 2021), PMCMRplus (Pohlert 2021), and lme4 (Bates et al. 2015).

## Results

### AMF root colonization

**Fig. 1:**
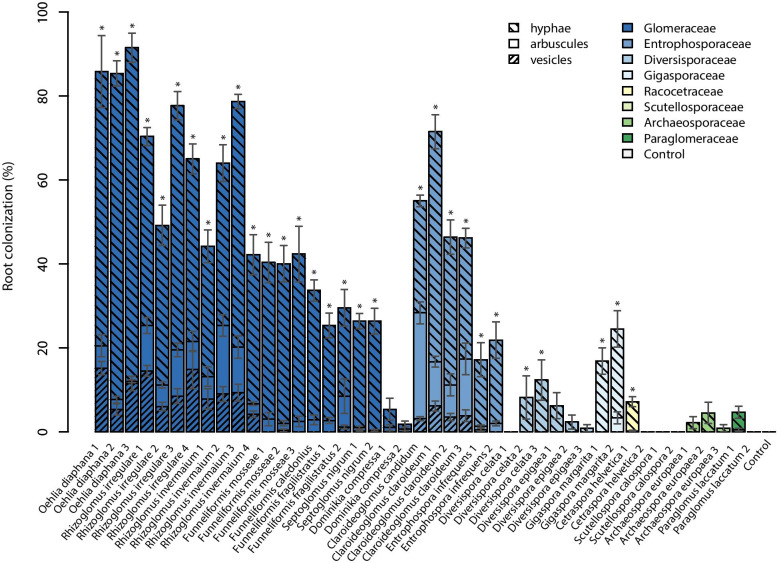
Intraradical hyphae, arbuscules, and vesicles summed to total root length colonization of leek plants inoculated with 44 different isolates of arbuscular mycorrhizal fungi (AMF) and one non-mycorrhizal control. Data are reported as means (*n* = 6) and their standard errors. Significant total root length colonization is indicated by asterisks and was determined with a beta regression model (*p* < 0.05)

Total AMF root colonization differed significantly among the 44 AMF isolates tested (Fig. 1 and Table S1). With the exception of *Dominikia compressa*, leek plants inoculated with AMF isolates of the families Glomeraceae and Entrophosporaceae (both belonging to the order Glomerales) had higher percentages of AMF root colonization than isolates of other families (Fig. 1). Isolates of *Oehlia*, *Rhizoglomus*, and *Claroideoglomus* had the highest percentages of AMF root colonization (on average 88% for *O. diaphana*, 66% for *R. irregulare*, and around 55% for *R. invermaium*, *Cl. candidum*, and *Cl. claroideum*). *Funneliformis*, *Septoglomus*, and *Entrophospora* spp. showed medium to high AMF root colonization (41% for *F. mosseae*, 34% for *F. caledonius*, 26% for *Septoglomus nigrum*, and 20% for *Entrophospora infrequens*), while *Dominikia compressa* had low colonization (4%). From the Diversisporaceae, *Di. celata* and *Di. epigaea* had only 7% and 3% AMF root colonization, respectively. Of the Gigasporaceae, *Gigaspora margarita* had 21%, and *Cetraspora helvetica* 4% AMF root colonization. AMF isolates of the Archaeosporaceae and Paraglomeraceae had low AMF colonization (3% for *Archaeospora europaea* and 2% for *Paraglomus laccatum*). No root colonization was observed in the control treatment without AMF added.

For some isolates, barely any colonization (isolate average of less than one percent) was detected, namely A.eur3, Ce.hel2, Di.cel1, Di.epi3, P.lac2, Sc.cal1, and Sc.cal2.

Linear regression analyses revealed no correlation between AMF spore densities of the AMF inocula and AMF root colonization (*r*^2^ = 0.03; *p* = 0.24; Fig. S1).

In the leek roots, vesicle formation was highest in *Oehlia*, *Rhizoglomus* and *Claroideoglomus* species (11% for *O. diaphana*, 12% for *R. irregulare*, 7% for *R. invermaium*, 5% for *Cl. claroideum*, and 3% for *Cl. candidum*). Vesicle formation was low to absent for other species of Glomeraceae and Entrophosporaceae, e.g., in *Funneliformis*, *Septoglomus*, *Dominikia*, and *Entrophospora* spp (Fig. 1). Vesicle formation was not observed for isolates of other AMF families.

Intraradical arbuscules were found in leek roots for all Glomeraceae and Entrophosporaceae species under study with the highest records for *Cl. candidum* (25%), *Cl. claroideum* (11%), *R. irregulare* and *R. invermaium* (each 9%), *F. fragilistratus* (5%), and *O. diaphana* (4%) and lowest in the *Dominikia*, *Septoglomus*, and *Entrophospora* isolates (c. 1%). For almost all other isolates, virtually no arbuscules were detected, with the exception of one *G. margarita* isolate (3%).

### Effects of AMF on plant biomass

**Fig. 2:**
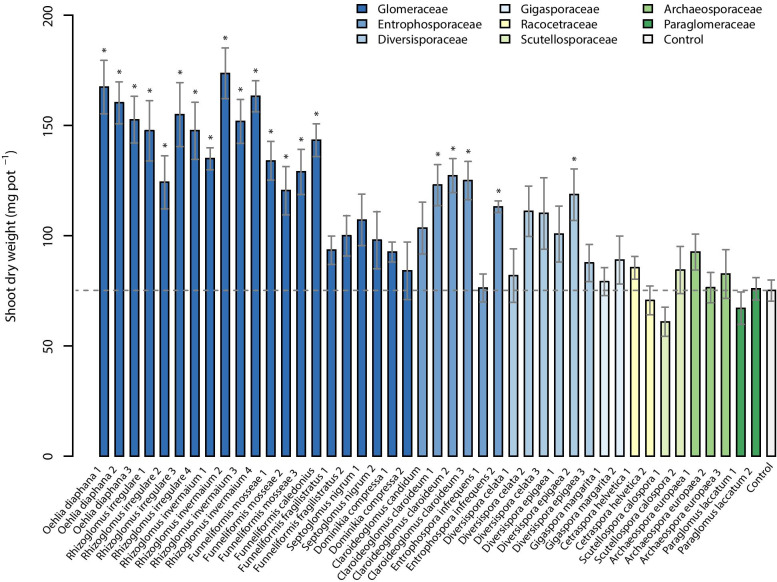
Aboveground biomass of leek inoculated with 44 different isolates of arbuscular mycorrhizal fungi (AMF) and one non-mycorrhizal control. Data are reported as means (*n* = 6) and their standard errors. Significant differences between AMF isolates and the control treatment (bar and dashed horizontal line) are indicated by asterisks and were determined with Dunnett’s test (*p* < 0.05) after a one-way ANOVA

**Fig. 3:**
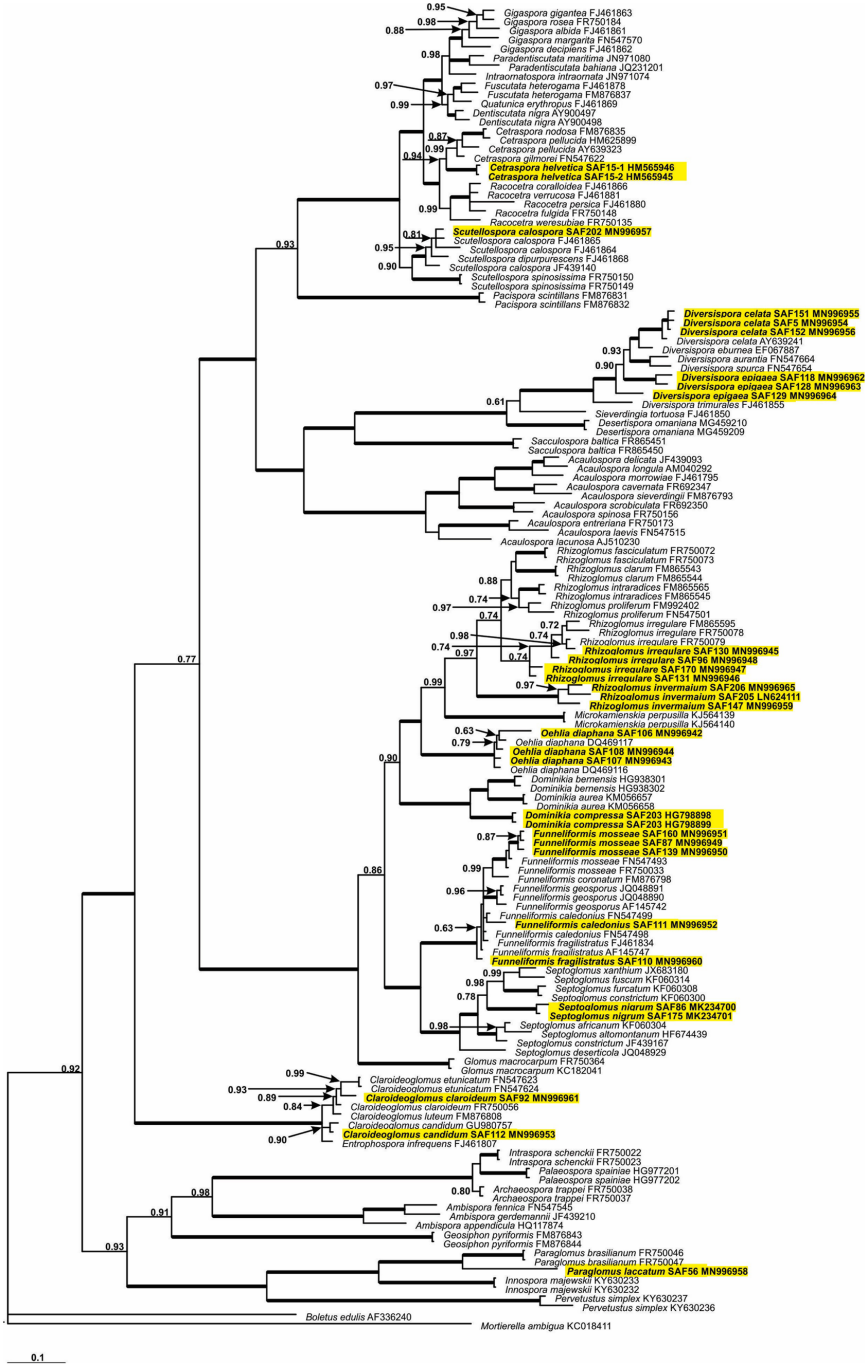
Phylogenetic tree of arbuscular mycorrhizal fungi (AMF) obtained by analysis of partial LSU rDNA sequences. The tree is based on the Bayesian Inference method. Sequences are labeled with their database accession numbers. Only support values of at least 60% are shown. Thick branches represent clades with 100% support. The tree was rooted by *Boletus edulis* and *Mortierella ambigua*. Isolates which are used for the inoculation experiment are marked in yellow (twelve isolates that could not be sequenced are missing)

Inoculation with 44 different AMF isolates clearly affected the shoot biomass production of the leek plants. Inoculation with 20 AMF isolates lead to significantly higher biomass than that of the controls (Fig. 2). None of the isolates had a statistically significant negative effect on leek shoot biomass. Overall, isolates with plant growth responses in similar magnitude belonged to the same taxonomic group (Fig. 2) and same phylogenetic clades, respectively (Fig. 3).

Results of restricted maximum likelihood analyses showed a highly significant effect of AMF species on leek biomass, but no significant effect of isolates within species (Table S2).

For species, the highest biomass production was observed for *Oehlia* and *Rhizoglomus* species (Fig. S2). For instance, the biomass of plants inoculated with *O. diaphana*, *R. invermaium*, and *R. irregulare* increased by 112%, 107%, and 91%, respectively, compared to the non-mycorrhizal controls. Also, after inoculation with *F. caledonius* (+ 90%), *F. mosseae* (+ 70%), and *Cl. claroideum* (+ 67%), leek plants produced significantly more biomass than the non-mycorrhizal controls. For other isolates from the families Glomeraceae and Entrophosporaceae (e.g., *Dominikia compressa*, *Septoglomus nigrum*, *Entrophospora infrequens*, and *Cl. candidum*); however, no significant differences versus the non-mycorrhizal control plants were detected. Although for three of the Diversisporales isolates, fairly high plant growth promotion was observed, these effects (+ 47 to + 57%) were not significant. Members of Gigasporales, Archaeosporales, and Paraglomerales showed no significant effect on leek biomass.

Significant effects were also found at the level of AMF genera (Fig. S3).

Multiple comparisons between AMF families showed that Glomeraceae had a significantly higher leek biomass than all other AMF families (+ 97 to + 17%) and than the control treatment (+ 76%; Fig. S4). Entrophosporaceae and Diversisporaceae also differed significantly from the control treatment (+ 50% and + 46%; Fig. S4).

**Fig. 4:**
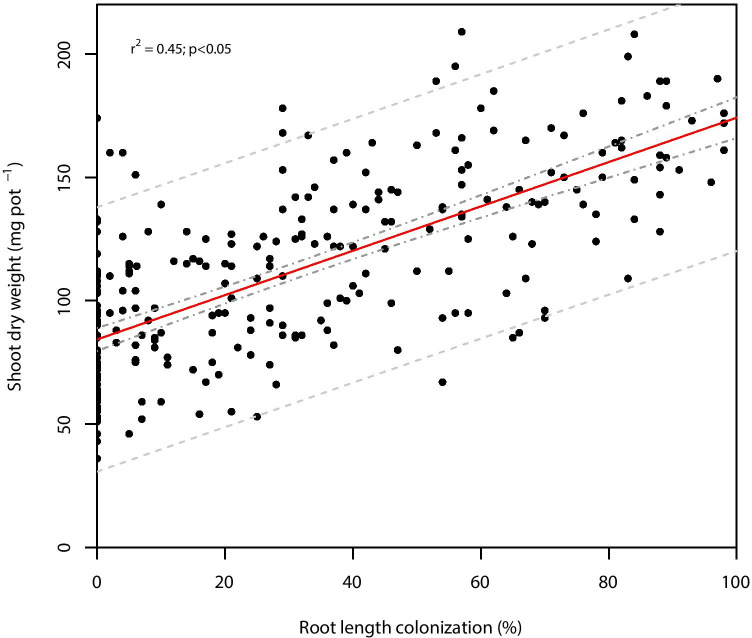
Linear regression between leek shoot dry weight and AMF root length colonization across all isolates (*r*^2^ = 0.45; *p* < 0.05) with 95% prediction (dashed lines) and confidence intervals (dash and dot lines)

Leek biomass was positively correlated with root length colonization (*r*^2^ = 0.45; *p* < 0.05; Fig. 4).

### Effects of AMF on nutrient concentrations in leek shoots

**Fig. 5:**
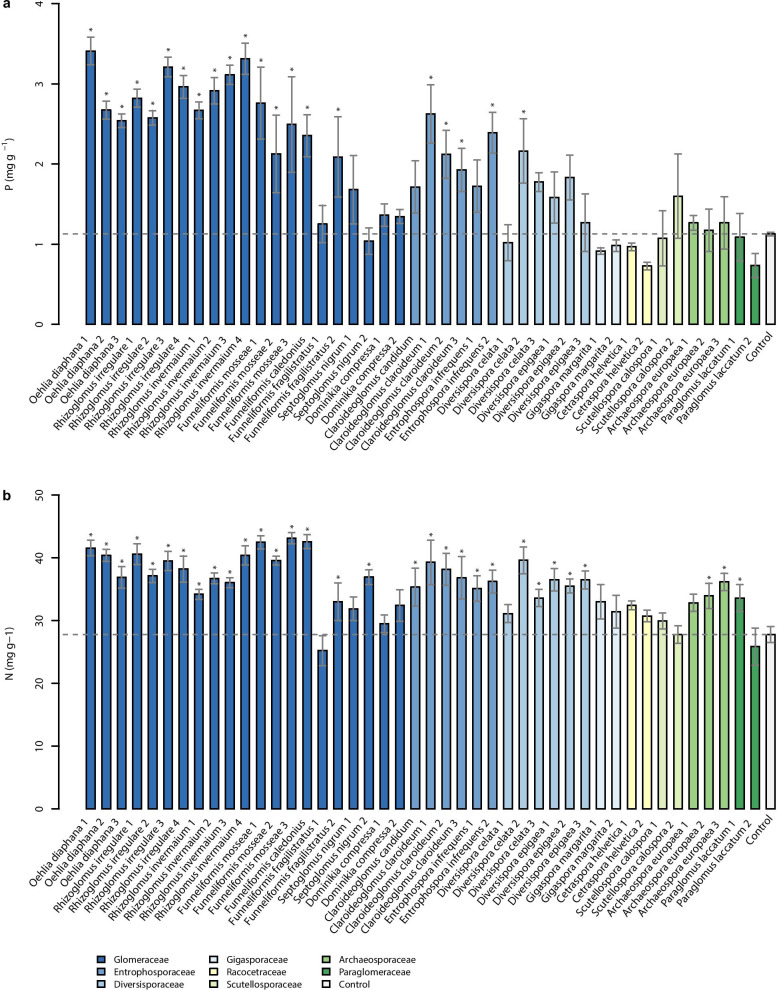
Phosphorus **a** and nitrogen **b** concentrations in above ground leek biomass inoculated with 44 different AMF isolates and one non-mycorrhizal control. Data are reported as means (*n* = 6) and their standard errors. Significant differences between AMF isolates and the control treatment (bar and dashed horizontal line) are determined with Conover’s many-to-one test (*p* < 0.05) after the non-parametric Kruskal–Wallis test and are indicated by asterisks

Phosphorus concentrations of the leek shoots were substantially affected by AMF inoculation. The effects of the AMF isolates on total P contents in leek plants were even stronger than those observed for shoot biomass. The phosphorus concentration in leek shoots was highest after inoculation with isolates of the species *O. diaphana* (+ 155% in average), *R. invermaium* (+ 166%), and *R. irregulare* (+ 156%; Fig. 5a). *Funneliformis* isolates led to elevated P concentrations; but these effects were not always significant. This also was found for *Claroideoglomus* and *Diversispora* isolates. Two species led to significantly increased P concentrations, namely *F. mosseae* and *Cl. claroideum*. There was no enhancement or a slight reduction of shoot P concentration after inoculation with *Do. compressa*, *Ce. helvetica*, and *Paraglomus laccatum* (Fig. 5a).

Similar to phosphorus assimilation, shoot nitrogen concentration was affected by AMF inoculation. The most effective isolates were F.mos3, F.cal, and F.mos1. There was no significant increase in shoot N concentration for a few Glomerales and Diversisporales isolates as well as for Ce.hel1 and P.lac1 (Fig. 5b). Taking the averages of the species, the shoot N concentration was highest after inoculation with *F. caledonius* (+ 53% when compared to the control treatment), followed by values obtained after inoculation with *F. mosseae* (+ 50%), *O. diaphana* (+ 43%), *R. irregulare* (+ 40%), *Cl. claroideum* (+ 37%), and *R. invermaium* (+ 33%).

N:P ratios of dry leek biomass ranged from 11.6 mgN/mgP (R.inv3) to 47.9 (Sc.cal1) mgN/mgP. Compared to the control treatment—which had a N:P ratio of 24.7—N:P ratios were significantly lower than the controls for all isolates of the genera *Oehlia* and *Rhizoglomus* as well as for the two isolates Cl.cla1 and E.inf2. Some isolates showed higher N:P ratios than controls, but with a high standard deviation of the means, effects were not significant (Fig. S5).

For some isolates—namely *Oehlia*, *Rhizoglomus*, *Claroideoglomus*, and *Diversispora* species—potassium concentration in leek aboveground biomass was slightly enhanced. In contrast, *Funneliformis* and *Paraglomus* isolates tended to result in a lower K concentration than that of the controls. However, these effects were not statistically significant (Fig. S6). Also, for magnesium concentration, there was no effect of AMF inoculation (Fig. S7). The majority of AMF isolates and species resulted in a lower calcium concentration of leek plants compared to the non-mycorrhizal controls (Fig. S8). The lowest Ca concentrations were found for all isolates of *Funneliformis* and *Septoglomus* with a decrease between 30% (Fu.mos1) and 36% (Fu.mos3).

## Discussion

This study demonstrates that different AMF taxa differ in their effects on plant growth. Especially ancient AMF lineages were least beneficial in terms of stimulating plant growth. The intraspecific differences among isolates of the same AMF species were small compared to those among AMF species. AMF species, genera and families displayed different performances in root colonization. Furthermore, we showed that taxonomic and phylogenetic characterization of AMF isolates based on morphology and molecular analysis were largely congruent. To obtain the best classification, it is recommended to apply both methods in combination (Oehl et al. 2011a).

Almost all AMF isolates that were tested showed a positive effect on leek biomass versus the non-mycorrhizal control. This was expected, because leek is known to be responsive to mycorrhizal fungi (e.g., Plenchette et al. 1982; Jansa et al. 2008; Karaca 2012; Kohler-Milleret et al. 2013). Some AMF isolates, however, resulted in higher growth promotion than other isolates. Such differences among AMF species in their ability to promote plant growth or nutrient acquisition have been observed multiple times (Jansa et al. 2008; Klironomos 2003; Pringle and Bever 2008; Tchabi et al. 2010; Thonar et al. 2011). Also, in the present study, different AMF species affected leek growth and nutrient uptake differently depending on the clade to which they belong. *Oehlia* and *Rhizoglomus* spp. provided the highest benefit to leek growth, followed by species belonging to *Funneliformis*, *Claroideoglomus*, and *Diversispora*, while Paraglomerales, Archaeosporales, and Gigasporales did not significantly affect leek growth.

From an evolutionary point of view, Paraglomerales and Archaeosporales are the most ancient AMF. These fungal clades date back about 460 million years, while members from the Glomerales and Gigasporales evolved more recently (Morton and Redecker 2001; Redecker et al. 2000a; Silva et al. 2012). A study by Koch et al. (2017) observed conservatism in fungal traits but did not find effects of fungal phylogeny on plant growth stimulation. In our study, however, species that diverged early in evolutionary history (e.g., *Paraglomus laccatum* and *Archaeospora* sp.; Redecker et al. 2000b) were less beneficial in stimulating plant biomass than phylogenetically younger taxa (especially Glomeraceae species). Different effects of ancient and more recently evolved AMF on plant biomass might be due to niche differentiation—e.g., members of the Paraglomeraceae have reduced root colonization and appear to be build extensive extra-radical mycelium in the soil (Hempel et al. 2007). Alternatively, the different effects might be due to differences in terms of carbon and nutrient exchange between plants and particular AMF taxa—e.g., ancient AMF appear to provide only limited amounts of nutrients to plants. As we did not assess extra-radical mycelium or carbon exchange, we only can hypothesize that these two factors may have caused differences in leek growth.

In a meta-analysis by Hoeksema et al. (2018), fungal phylogeny and fungal identity (genus) did not explain plant responses to AMF. Besides context dependency, this could be due to few studies actually having tested the response of plants to ancient AMF and very few data entries for ancient AMF taxa being included in the database making it difficult to test this. Additionally, our study was unbalanced in that only a few isolates of *Paraglomus* and *Archaeospora* were tested, making these ancient AMF taxa underrepresented. Nevertheless, in this study, we analyzed clearly more “ancient” AMF taxa than did preceding works. It also should be taken into account that our study reflects the natural distribution of AMF genera, as the current classification of AMF accepts 20 genera in the order Glomerales, while there are only ten in Diversisporales, eleven in Gigasporales, five in Archaeosporales and three in Paraglomerales (Wijayawardene et al. 2020). This indicates that there simply are fewer ancient species available. Sieverding and Howeler (1985) and Howeler et al. (1987) evaluated a range of AMF species, including the ancient species *Paraglomus occultum* and found varying effects of this species, ranging from no effect to significant plant growth promotion. Powell et al. (2009) examined 27 AMF species and assessed with compiled data from three different glasshouse studies whether AMF phylogeny is related to functional traits of AMF (root and soil colonization, plant growth benefit). They observed that some traits, like root and soil colonization or host benefits, may be phylogenetically conserved with which our study agrees.

Hart and Reader (2002a) showed that in two plant species (*Plantago lanceolata* and *Poa annua*) Glomeraceae provided the greatest and Acaulosporaceae the least plant growth promotion. They related this to the large internal mycelium of Glomeraceae. Differences in carbon demand (Elbon and Whalen 2015) also are considered. Tchabi et al. (2010) showed that also several *Acaulospora* species derived from tropical areas, however, might provide significant plant growth promotion under tropical conditions, and that species such as *A. scrobiculata*, *A. minuta*, and *A. spinosissima*, might also be able to elaborate substantial internal mycelium (Tchabi et al. 2010; Oehl et al. 2014a, b).

Because our experiment was run only for 8 weeks from plant emergence, life cycles of the symbionts may play a significant role in the positive effects on plant growth. Life cycles of *Rhizoglomus*, *Funneliformis*, *Oehlia*, and *Claroideoglomus* species are faster than those of *Dominikia* and *Septoglomus* species and Gigasporales species in the temperate zones (Oehl et al. 2009). These observations do not explain the ineffectiveness of the *Archaeospora* and *Paraglomus* isolates, which have similarly fast life cycles to *Oehlia diaphana*, *Funneliformis mosseae*, *Rhizoglomus irregulare*, *Claroideoglomus claroideum*, and *Cl. etunicatum* (Oehl et al. 2005, 2009). Nevertheless, there are differences in colonization rate, i.e., the time from inoculation to the first colonization of roots. In a screening of 21 AMF isolates by Hart and Reader (2002b), species of Glomeraceae were found to be the fastest in root colonization with an initial root colonization from 1 to 3 weeks after inoculation depending on the species, while some members Gigasporaceae and Acaulosporaceae needed 6 to 8 weeks. This may be one reason—among others—that in our experiment, inoculation with isolates of Glomeraceae resulted in highest leek biomass gain.

Another aspect is the cultivation duration of isolates. Although all AMF isolates were propagated under same conditions, some isolates—namely Di.cel1, all isolates of Gigasporales and Paraglomerales—were propagated for a longer time than the others. Bentivenga et al. (1997) showed that propagation can change characteristics such as spore size and color in a few generations. We cannot exclude that interaction traits of AMF with plants changed over the time in pot culture.

Some studies found functional variation within AMF species (e.g., de Novais et al. 2014; Koch et al. 2006; Munkvold et al. 2004). In our study, however, the variation among isolates of the same species was less pronounced than that among species. This probably is because the inocula of different isolates of the same species had similar spore densities, identical ages and were obtained in similar conditions. Therefore, they probably were of very similar quality which could explain why intra-specific variability was low in our study.

Overall, AMF isolates which increased leek biomass enhanced shoot P and N concentrations as well. Previous studies also showed the effect of AMF accumulating P and/or N in plant biomass for leek (Fusconi et al. 2005; Hart and Forsythe 2012) and for other plant species such as tomato (Ortas et al. 2013), *Medicago truncatula* (Burleigh et al. 2002), *M. sativa* (Avio et al. 2006), and apple (Cavallazzi et al. 2007). Lendenmann et al. (2011) suggested that differences in P acquisition between *R. intraradices* and *Cl. claroideum* are due to differences in mycelium length density and in P transporters. In addition, there also is evidence that Gigasporaceae can retain P in their hyphae before it is transported to the host plant (Jakobsen et al. 1992; Dodd et al. 2000; Solaiman and Saito 2001). Even if there is no growth response by an AMF inoculated plant, P uptake via the mycorrhizal pathway can occur; hence, the fungus contributes to the plant’s nutrition (Smith et al. 2004). Thus, under experimental conditions with P as the growth-limiting factor, optimal plant growth promotion can be attained by AMF that provide the greatest P transfer to the host plant with the least demand for carbon (Burleigh et al. 2002).

Güsewell (2004) stated that plants with N:P ratios < 10 often are N-limited in biomass production, while plants with N:P ratios > 20 instead are limited by phosphorous. More than half of the AMF isolates of the present study resulted in a N:P ratio over 20, indicating that those treatments probably were P limited. There was no N:P ratio below 10, so we suppose that none of the leek plants lacked N. Those isolates that led to low N:P ratios also are those that resulted in elevated plant biomass. Therefore, we conclude that growth promotion by those isolates was most likely because they improved P supply.

While the acquisition of most nutrients was positively influenced (N and P) or not affected (K and Mg) by AMF, Ca concentrations were notably low in the control treatment. This also was observed by Baslam et al. (2011). They attributed this to a dilution effect because inoculated plants showed elevated biomass. In our case, plants with no growth response also had low Ca concentrations; therefore, we think this does not apply for our case. As we did not assess Ca concentrations in roots, we cannot identify if uptake from the soil via roots or via the AMF pathway is reduced, or if Ca is retained in the roots of AMF inoculated leek plants.

In our study, root colonization was positively correlated with biomass gain with species of Archaeosporaceae and Paraglomeraceae having the lowest colonization and plant growth responses. As expected, vesicle formation by Gigasporales, Archaeosporales, and Paraglomerales isolates was not observed. Two Diversisporales species also formed no vesicles in contrast to earlier reports (Oehl et al. 2011b; Błaszkowski 2012; Błaszkowski et al. 2019). It must be considered that hyphae, arbuscules, and vesicles of Archaeosporaceae and Paraglomeraceae barely stain in trypan blue or ink (Oehl et al. 2011a, b) and therefore might have been underestimated. For sparsely colonizing isolates, it also is possible that the establishment of the symbiosis was not successful. Previous studies show divergent results. Intense fungal root colonization often is not associated with improved plant growth (Burleigh et al. 2002; Mensah 2015; Munkvold et al. 2004; Smith et al. 2004; Tchabi et al. 2010). Avio et al. (2006) showed a positive correlation between these two factors, however, and Treseder (2013) revealed in a meta-analysis that plant growth generally tends to improve with increased root colonization.

Early plants did not have extensive root systems and therefore they likely were limited in their uptake of mineral nutrients (Fitter 2005). As plants developed extensive, complex root systems, AMF co-evolved. Hence, plants provided an important habitat for AMF which became dependent on their host plants, while plants became more efficient in mycorrhiza formation (Brundrett 2002). Thus, the reduced ability of ancient AMF to promote nutrient uptake and plant growth might be related to limited root colonization.

There might be other factors than taxonomic and phylogenetic traits of AMF that determine the beneficial effects of AMF species on leek growth. Gigasporales species are known to be frequent in warm climates and thus may be inefficient for leek in cool/temperate climates than better adapted clades such as *Funneliformis*, *Rhizoglomus* or *Oehlia* (Oehl et al. 2017). From our results, we conclude that taxonomic relatedness of AMF species likely can be used to make predictions about functional relationships in the symbiosis between plants and AMF. In our study, however, only a few ancient isolates like *Paraglomus laccatum* were available and our dataset was biased towards lineages of Glomeraceae. Therefore, these results may not provide a complete picture. We highly recommend integration of additional ancient species in future tests.

## Supplementary Information

Below is the link to the electronic supplementary material.

Supplementary file1 (PDF 1138 KB)

## Data Availability

DNA sequences are deposited in the NCBI GenBank (www.ncbi.nlm.nih.gov/genbank/). Other data generated during the current study are available from the corresponding author.
