# Supplementary material for: Ancient lineages of arbuscular mycorrhizal fungi provide little plant benefit
Source: Mycorrhiza. 2021 Jul 30;31(5):559–76. doi: 10.1007/s00572-021-01042-5 (PMC8484173; doi:10.1007/s00572-021-01042-5)
Supplement: Supplementary file 1 — Supplementary file1 (PDF 1138 KB) [file 572_2021_1042_MOESM1_ESM.pdf]

Ancient lineages of arbuscular mycorrhizal fungi provide little plant benefit

Supplementary Material

Fig. S1 Regression analyses for AMF spore numbers of inocula and AMF root colonization of leek plants (r^2 =0.03).

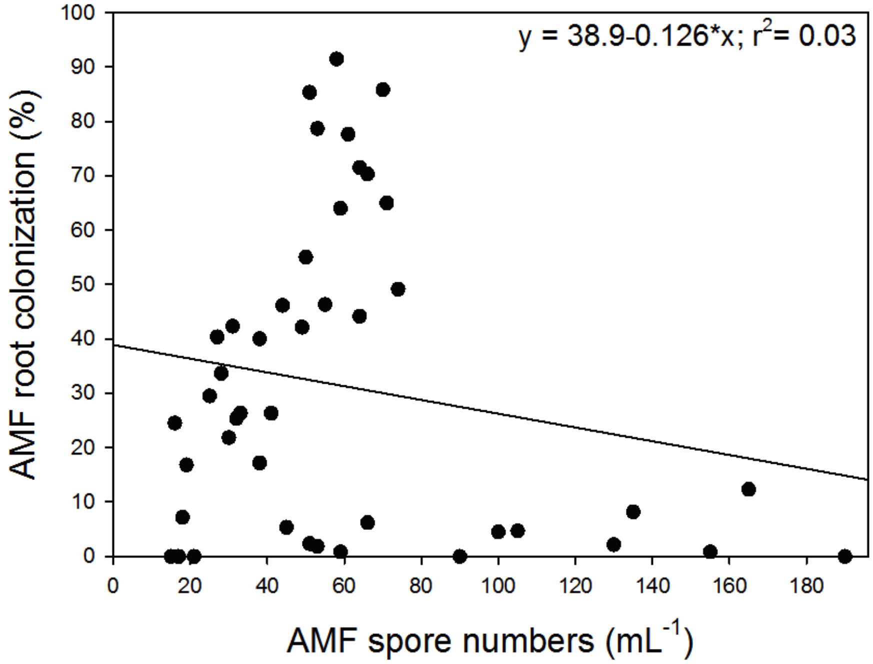

Fig. S2 Impact of different AMF species on leek biomass. Data are reported as means and their standard error. Significant differences between AMF isolates and the control treatment (bar and dashed horizontal line) are indicated by asterisks and were determined with Dunnett's test ($p < 0.05$) after a one-way ANOVA

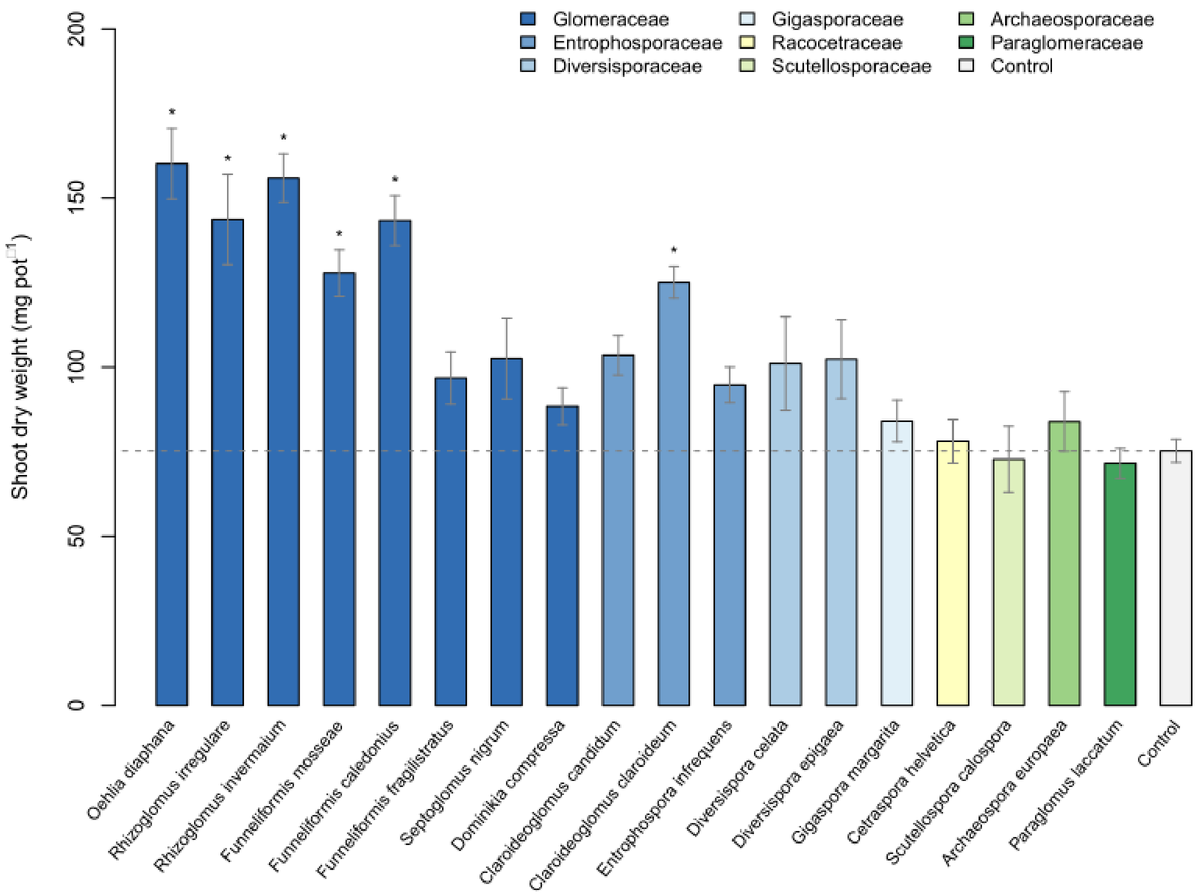

Fig. S3 Impact of different AMF genera on leek biomass. Data are reported as means and their standard error. Significant differences between AMF isolates and the control treatment (bar and dashed horizontal line) are indicated by asterisks and were determined with Dunn's test ($p < 0.05$) after Kruskal-Wallis test

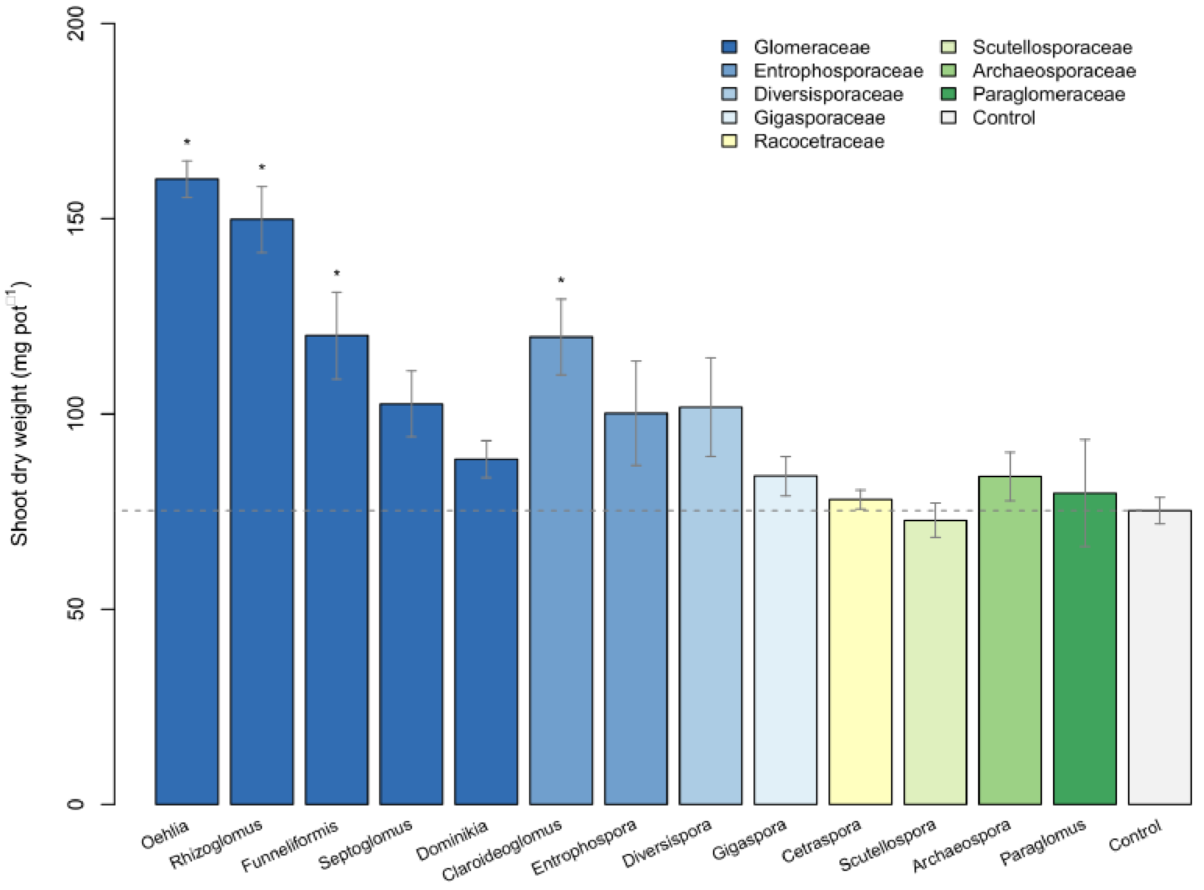

Fig. S4 Impact of different AMF families on leek biomass. Data are reported as means and their standard error. Significant differences between AMF isolates and the control treatment (bar and dashed horizontal line) are indicated by asterisks and were determined with Dunn's test ($p < 0.05$) after Kruskal-Wallis test.

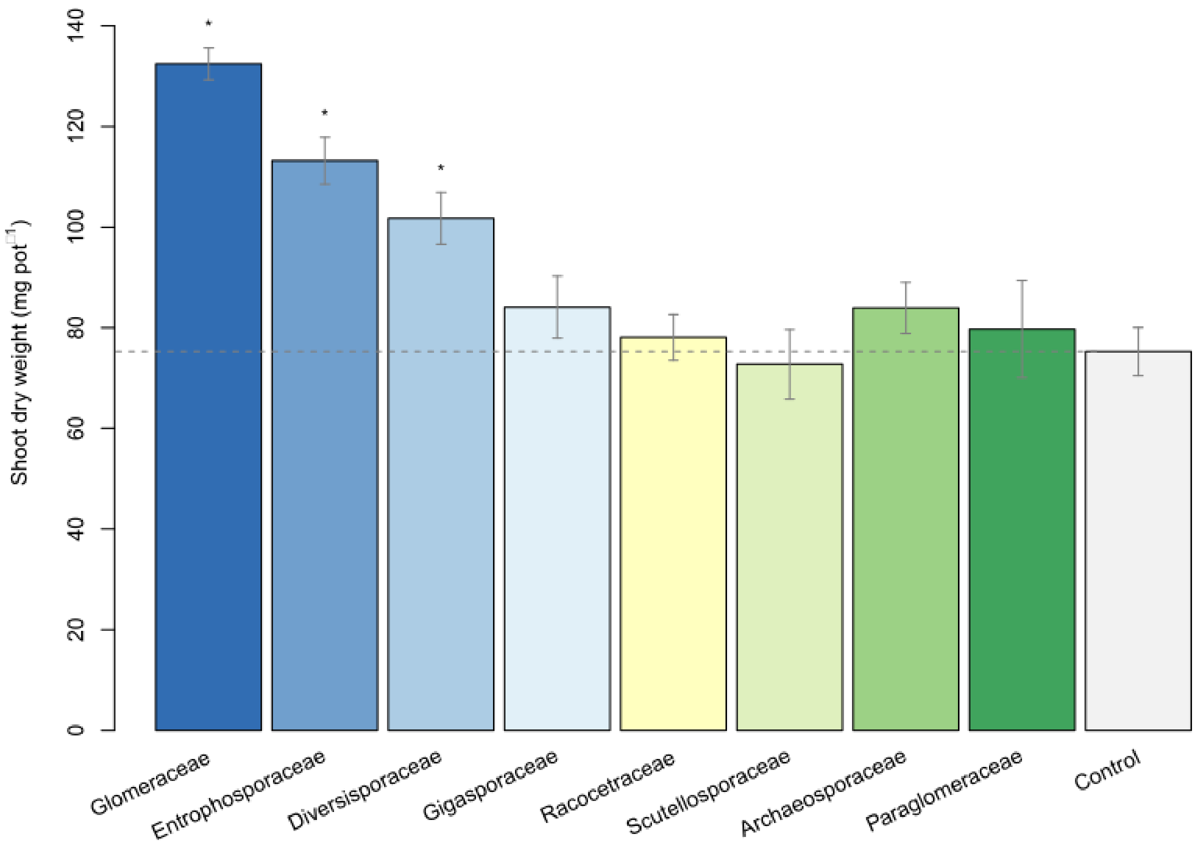

Fig. S5 N:P ratios in above ground leek biomass inoculated with 44 different AMF isolates and one non-mycorrhizal control. Data are reported as means (n=6) and their standard error. Significant differences between AMF isolates and the control treatment (bar and dashed horizontal line) are determined with Conover's many-to-one test ($p<0.05$) after the non-parametric Kruskal-Wallis test and indicated by asterisks.

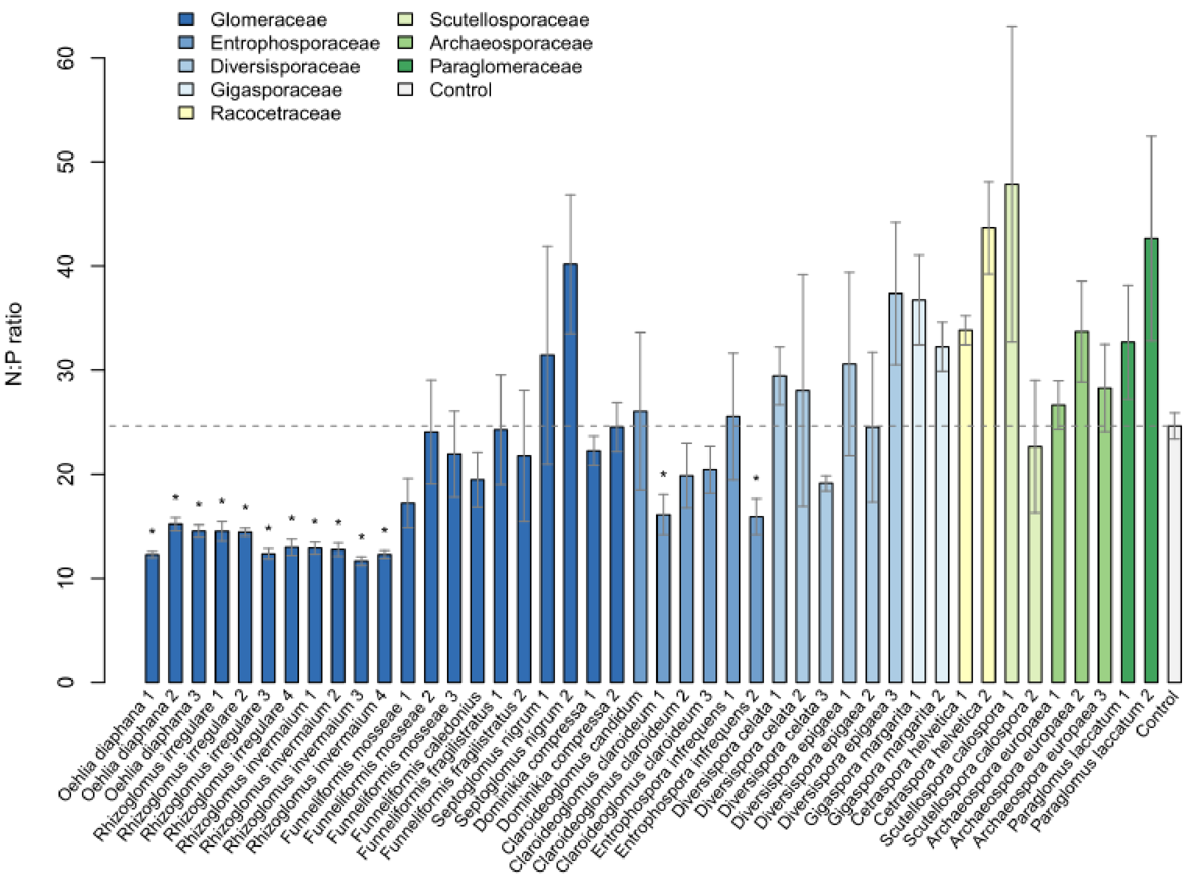

Fig. S6 Potassium concentration in the shoot biomass inoculated with 44 different AMF isolates and one non-mycorrhizal control. Data are reported as means (n=6) and their standard error. Significant differences between AMF isolates and the control treatment (bar and dashed horizontal line) are determined with Dunn's test ($p<0.05$) after the non-parametric Kruskal-Wallis test and indicated by asterisks

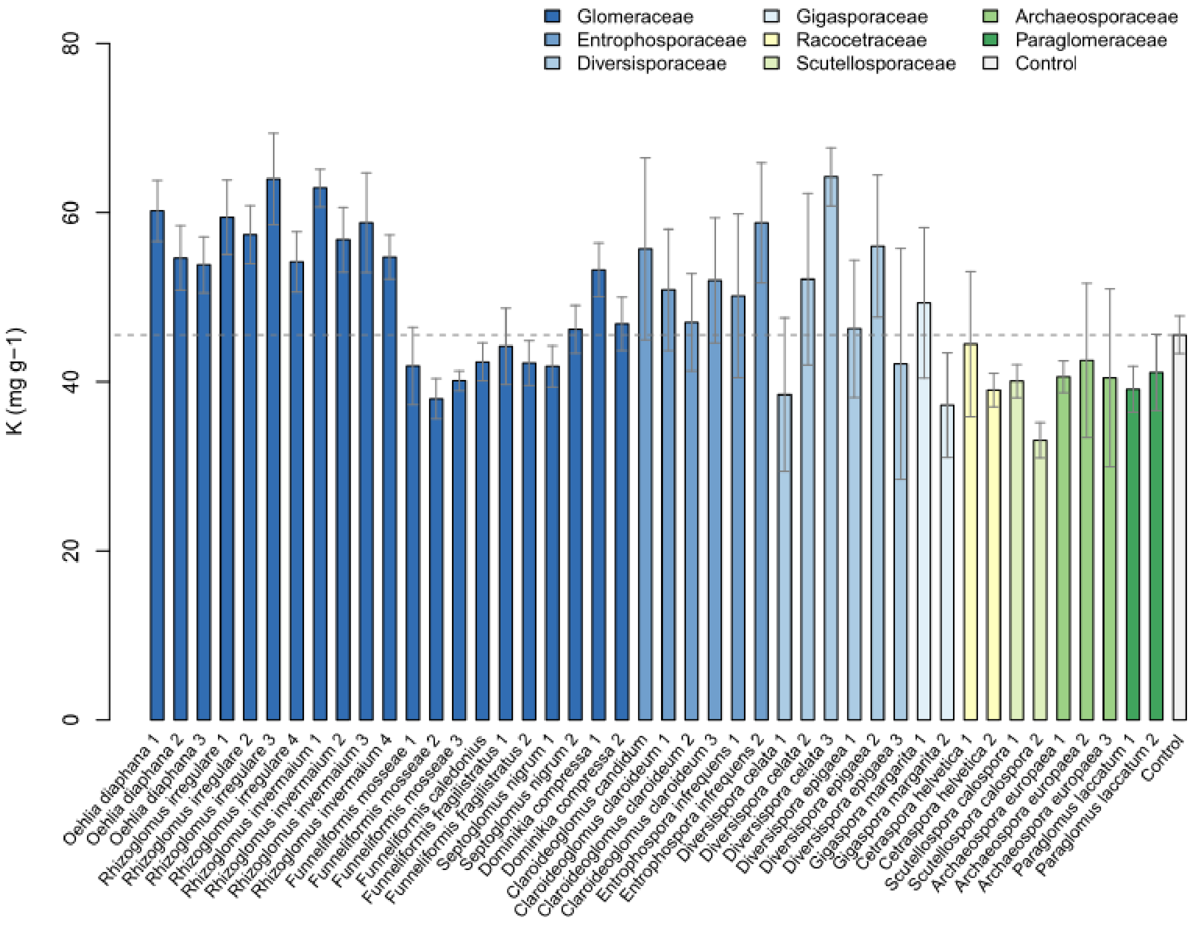

Fig. S7 Magnesium concentration in the shoot biomass inoculated with 44 different AMF isolates and one non-mycorrhizal control. Data are reported as means (n=6) and their standard error. Significant differences between AMF isolates and the control treatment (bar and dashed horizontal line) are determined with Dunn's test (p<0.05) after the non-parametric Kruskal-Wallis test and indicated by asterisks

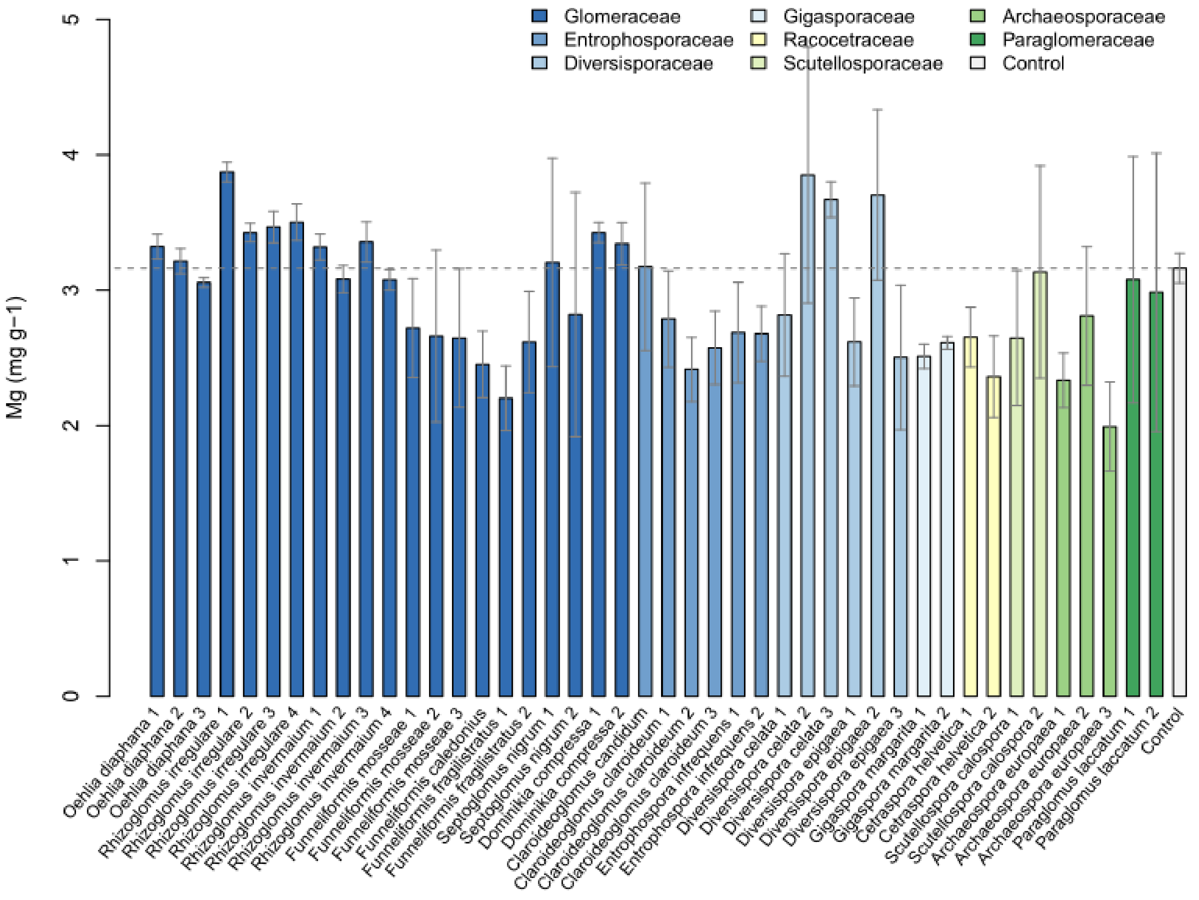

Fig. S8 Calcium concentration in the shoot biomass inoculated with 44 different AMF isolates and one non-mycorrhizal control. Data are reported as means (n=6) and their standard error. Significant differences between AMF isolates and the control treatment (bar and dashed horizontal line) are determined with Dunn's test ($p<0.05$) after the non-parametric Kruskal-Wallis test and indicated by asterisks

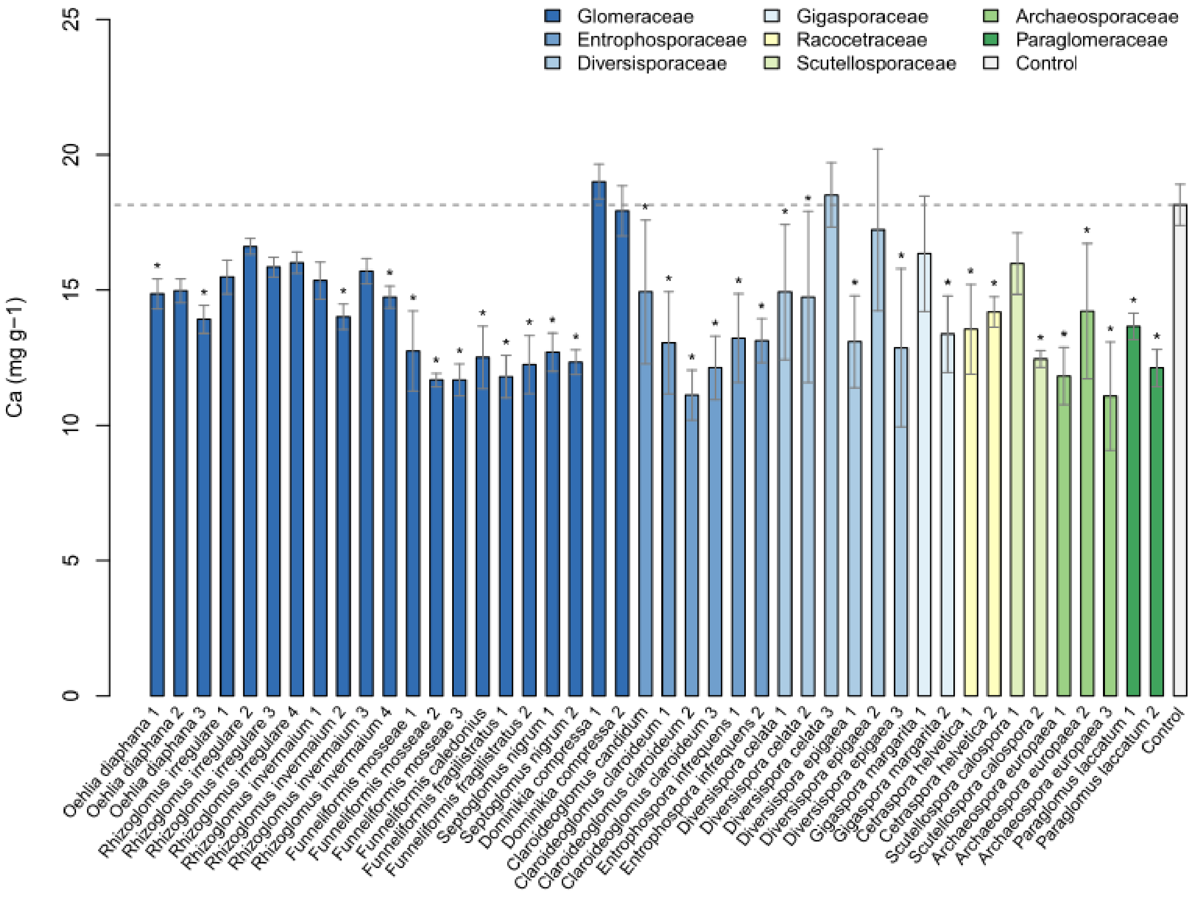

Table S1 Total AMF root colonization and presence of vesicles and arbuscules in leek roots (in %) ten weeks after inoculation.

Isolate	AMF root colonization mean per		Vesicles mean per		Arbuscules mean per	
	isolate	species	isolate	species	isolate	species
O.dia1	85.8*	87.5*	15.2*	10.7*	5.3*	3.8*
O.dia2	85.3*		5.3*		2.3	
O.dia3	91.5*		11.5*		0.5	
R.irr1	70.3*	65.6*	14.5*	7.8*	10.8*	9.0*
R.irr2	49.2*		0.3*		5.2*	
R.irr3	77.7*		8.5*		11.0*	
R.irr4	65.0*		14.8*		6.7*	
R.inv1	44.2*	55.0*	7.8*	5.4*	5.3*	8.7*
R.inv2	64.0*		0.4*		16.3*	
R.inv3	78.7*		9.3*		10.8*	
R.inv4	42.2*		4.2*		2.5*	
F.mos1	40.3*	40.9*	0.0	0.1	3.0	2.4
F.mos2	40.0*		0.3		1.7	
F.mos3	42.3*		0.0		2.5	
F.cal	33.7*	33.7*	0.0	0.0	2.8	2.8
F.fra1	25.3*	27.4*	0.0	0.6	2.7	5.0*
F.fra2	29.5*		1.2		7.3	
Se.nig1	26.3*	26.3*	0.2	0.1	0.8	0.6
Se.nig2	26.3*		0.0		0.3	
Do.com1	5.3	3.6	0.0	0.0	1.0	0.9
Do.com2	1.8		0.0		0.7	
Cl.can	55.0*	55.0*	3.2*	3.2*	25.2*	25.2*
Cl.cla1	46.2*	54.7*	3.9*	4.5*	13.5*	10.6*
Cl.cla2	71.5*		6.2*		10.5*	
Cl.cla3	46.3*		3.5*		7.7*	
E.inf1	17.2*	19.5*	0.5	0.3	0.8	1.4
E.inf2	21.8*		0.0		2.0	
Di.cel1	0.0		0.0		0.0	
Di.cel2	8.2	10.3	0.0	0.0	0.0	0.0
Di.cel3	12.3*		0.0		0.0	
Di.epi1	6.2	3.1	0.0	0.0	0.0	0.0
Di.epi2	2.3		0.0		0.0	
Di.epi3	0.8		0.0		0.0	
G.mar1	16.8*	20.7*	0.0	0.0	0.0	1.7
G.mar2	24.5*		0.0		3.3	

Ce.hel1	7.2	3.6	0.0	0.0	0.2	0.1
Ce.hel2	0.0		0.0		0.0	
Sc.cal1	0.0	0.0	0.0	0.0	0.0	0.0
Sc.cal2	0.0		0.0		0.0	
A.eur1	2.2	2.5	0.0	0.0	0.0	0.0
A.eur2	4.5		0.0		0.0	
A.eur3	0.8		0.0		0.0	
P.lac1	4.7	2.4	0.5	0.3	0.0	0.0
P.lac2	0.0		0.0		0.0	
Control	0.0		0.0	0.0	0.0	0.0

*significant at $p<0.05$ determined by Fischer’s LSD-test

Table S2 REML analyses for the effect of AMF species and AMF isolates within species on leek biomass.

Variable	Sum of squares	d. f.	F-value	p-value
(Intercept)	43 861	1	62.04	< 0.0001***
Species	73 319	14	7.41	< 0.0001***
Isolates within species	10 579	23	1.15	0.322
Residuals	98 979	140		
